# Origin of 1/*f* noise in hydration dynamics on lipid membrane surfaces

**DOI:** 10.1038/srep08876

**Published:** 2015-03-06

**Authors:** Eiji Yamamoto, Takuma Akimoto, Masato Yasui, Kenji Yasuoka

**Affiliations:** 1 Department of Mechanical Engineering, Keio University, 3-14-1 Hiyoshi, Kohoku-ku, Yokohama 223-8522, Japan; 2 Department of Pharmacology, School of Medicine, Keio University, 35 Shinanomachi, Shinju-ku, Tokyo 160-8582, Japan

## Abstract

Water molecules on lipid membrane surfaces are known to contribute to membrane stability by connecting lipid molecules and acting as a water bridge. Although water structures and diffusivities near the membrane surfaces have been extensively studied, hydration dynamics on the surfaces has remained an open question. Here we investigate residence time statistics of water molecules on the surface of lipid membranes using all-atom molecular dynamics simulations. We show that hydration dynamics on the lipid membranes exhibits 1/*f* noise. Constructing a dichotomous process for the hydration dynamics, we find that residence times in each state follow a power-law with exponential cutoff and that the process can be regarded as a correlated renewal process where interoccurrence times are correlated. The results imply that the origin of the 1/*f* noise in hydration dynamics on the membrane surfaces is a combination of a power-law distribution with cutoff of interoccurrence times of switching events and a long-term correlation between the interoccurrence times. These results suggest that the 1/*f* noise attributed to the correlated renewal process may contribute to the stability of the hydration layers and lipid membranes.

In numerous natural systems, the power spectra *S*(*f*) exhibit enigmatic 1/*f* noise:
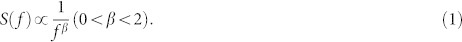
at low frequencies. In biological systems, 1/*f* noise has been reported for protein conformational dynamics^1^,^2^,^3^, DNA sequences^4^, biorecognition^5^, and ionic currents^6^,^7^,^8^,^9^, implying that long-range correlated dynamics underlie biological processes. In particular, 1/*f* noise is involved in the regulation of permeation of water molecules in an aquaporin^3^.

There are many mathematical models that generate 1/*f* noise including stochastic models^10^,^11^,^12^,^13^ and intermittent dynamical systems^14^,^15^,^16^,^17^. The power-law residence time distribution is one of the most thoroughly studied origins for 1/*f* noise^12^,^14^,^15^,^16^,^17^. In dichotomous processes, the power spectrum shows 1/*f* noise when the distribution of residence times of each state follows a power-law distribution with divergent second moment. For blinking quantum dots, which show a 1/*f* spectrum, residence times for “on” (bright) and “off” (dark) states have been experimentally shown to have a power-law distribution with a divergent mean^18^,^19^. In stochastic models, this divergent mean residence time violates the law of large numbers which causes the breakdown of ergodicity, non-stationarity, and aging^20^,^21^,^22^,^23^,^24^,^25^. On the other hand, the divergent mean residence time implies an infinite invariant measure in dynamical systems^26^ and that the time-averaged observables are intrinsically random^26^,^27^.

Water structures and diffusivities near lipid membrane surfaces have been extensively studied^28^,^29^,^30^,^31^,^32^,^33^,^34^,^35^,^36^,^37^,^38^. Orientations of water molecules are aligned on the surfaces heterogeneously^32^,^37^. The complex structure and viscoelasticity of the lipid membranes cause anomalous diffusion^34^,^35^,^36^ and aging phenomena^36^ of water molecules on the surfaces. In our previous work, we found that the residence times of water molecules on the lipid membrane surfaces follow power-law distributions^33^,^36^. Therefore, it is physically reasonable to expect that the hydration dynamics on membrane surfaces also obey 1/*f* noise. Although little is known about the hydration dynamics, it is important to understand the dynamics of resident water molecules because these water molecules may play important roles in the overall dynamics of the membrane, and will affect membrane stability and biological reactions. In fact, such water molecules stabilize the assembled lipid structures^30^; this water retardation increases the efficiency of biological reactions^39^,^40^. Water molecules enter and exit the hydration layer, and the number of water molecules near the lipid head group fluctuates.

Here, we perform a molecular dynamics (MD) simulation on water molecules plus pure palmitoyl-oleoyl-phosphocholine (POPC) membrane at 310 K to investigate the hydration dynamics on the lipid surface. We find that fluctuations in the number of water molecules on the lipid surface show 1/*f^β^* noise with *β* > 1 at high frequencies, and that the residence time distributions for “on” and “off” states follow power-law distributions with exponential cutoffs. Moreover, we construct a dichotomous process from the trajectory of the number of water molecules on a lipid molecule to clarify the origin of the 1/*f* noise. By analyzing the constructed dichotomous process, we find that there is a long-term correlation between residence times, which contributes to the *β* > 1 at high frequencies.

## Results

### Fluctuations of water molecules on the lipid head group

**Figure 1:**
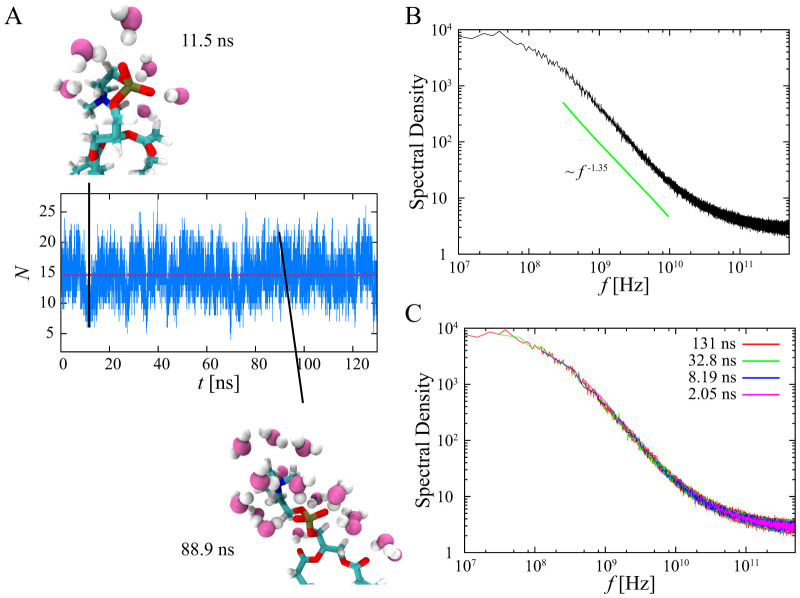
Power spectrum of the number of water molecules on the POPC membrane. (A) Time series of number of water molecules on a lipid head group. The red dashed line is the average number of water molecules on the lipid head group over this time period. The outer windows show snapshots of water molecules around the lipid head group. (B) Ensemble-averaged PSD of number of water molecules. We use 128 time series to obtain the ensemble-averaged PSD. The solid lines represent power-law behavior for reference. Total measurement time was 131 ns. (C) Ensemble-averaged PSD for four different measurement times: 2.05, 8.19, 32.8, and 131 ns. The power spectra coincide without fitting.

To investigate the hydration dynamics on the lipid membrane surfaces, we recorded the number of water molecules for which the oxygen was within interatomic distances of 0.35 nm^38^,^41^, which corresponds to the hydrogen bond distance, from all atoms in lipid head group [Fig. 1A]. The number fluctuates around the average of about 14. Figure 1B shows the ensemble-averaged power spectra density (PSD) of the water molecules calculated for the 128 lipid molecules in the POPC lipid bilayer. We have *S*(*f*) ∝ *f*^−*β*^ with *β* = 1.35 ± 0.05 at high frequencies, while below a transition frequency (*f_t_* = 0.3 GHz) PSD becomes a plateau at low frequencies. This crossover phenomenon is essential because *S*(*f*) ∝ *f*^−*β*^ with *β* ≥ 1 implies non-integrability and non-stationarity. The time scale of the 1/*f* noise is longer than 100 ps (*f* = 10^10^ Hz). Mean residence time of water molecules on the membrane surface is 7–71 ps depending on the biding sites and definitions of hydrated molecules^30^,^31^,^36^,^38^, which is shorter than the time scale of the 1/*f* noise. Moreover, 80% of water molecules which continuously reside on the membrane surface more than 1 ns move beyond 0.6 nm^2^ 36. The area per lipid is about 0.5–0.7 nm^2^. Thus, most of the water molecules are displaced on the membrane surface by exchanging hydrogen bond interactions with lipid molecules in the time scale of the 1/*f* noise. We have confirmed that fluctuations of the number of water molecules within a box and a sphere near the membrane surfaces also exhibit 1/*f* noises but this is not the case for bulk [see Fig. S1].

A similar transition of the power-law exponent of the PSD has also been observed for the interchange dynamics of “on” and “off” states for quantum dot blinking^42^. This behavior was described theoretically using an alternating renewal process, where the residence time distributions of “on” and “off” states are given by a power-law with an exponential cutoff 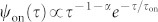 and a power-law *ψ*_off_(*τ*) ∝ *τ*^−1−*α*^ with *α* < 1, respectively^42^. The transition frequency *f_t_* is related to the exponential cutoff in the quantum dot blinking experiment. In this case, the PSD exhibits aging, non-stationarity, and weak ergodicity breaking because the “off” time does not have a finite mean. To confirm whether the aging effect appears in the hydration dynamics on the lipid surface, we calculate the ensemble-averaged PSDs for different measurement times [Fig. 1C]. The magnitudes of the PSDs do not depend on the measurement time *t*, *i.e.* there is no aging. It follows that the power-law distribution with an exponential cutoff considered in the quantum dot experiment^42^ cannot explain hydration dynamics on the lipid membrane surfaces.

### Origin of the 1/*f* noise

**Figure 2:**
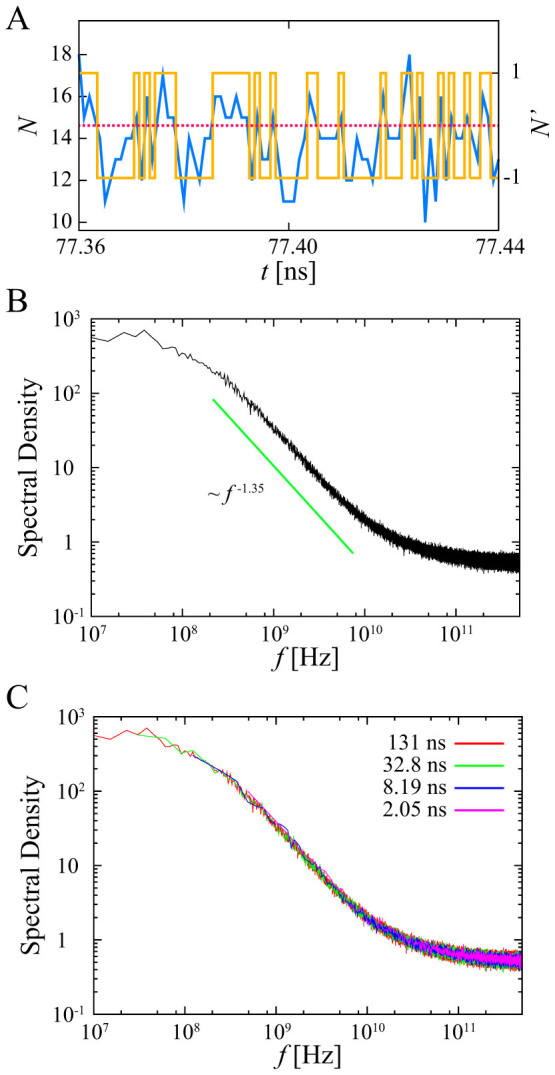
1/*f* noise in the dichotomous process on the POPC membrane. (A) Part of a time series of the number of water molecules on a lipid molecule (blue line); conversion of this data into “on” or “off” states (yellow line), depending on whether the number of water molecules is above or below the average (red dashed line). (B) Ensemble-averaged PSD of the dichotomous process. The solid line is shown as reference. (C) Ensemble-averaged PSD of the time series of the two states for four different measurement times: 2.05, 8.19, 32.8, and 131 ns. There is no aging.

One important question remains unclear: What is the origin of the 1/*f* noise? In other words, does power-law intermittency or long-term memory contribute to the 1/*f* noise? To consider the origin of 1/*f* noise, we constructed a dichotomous (two states) process from the time series of the number of water molecules, where a state is called “on” (*N*′ = 1) state when the number of water molecules on each lipid molecule is above the average number and “off” (*N*′ = −1) state otherwise [Fig. 2A]. Figure 2B shows the ensemble-averaged PSD for the time series of constructed dichotomous processes. The obtained 1/*f* noise is the same as the ensemble-averaged PSD for the original time series [see Fig. 1B]. The PSD of the dichotomous processes also does not show aging [Fig. 2C].

**Figure 3:**
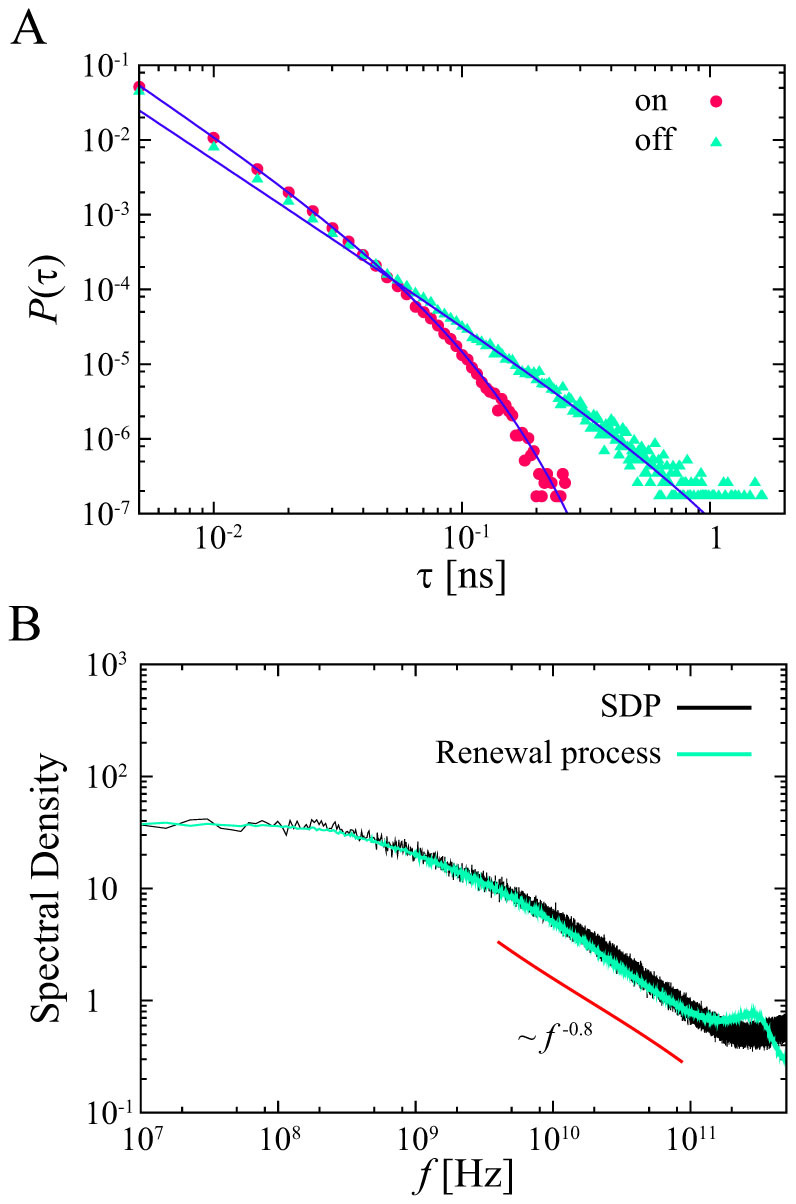
Alternating renewal process. (A) PDFs of residence times of “on” and “off” states. Solid lines are fitted curves for power-law distributions with exponential cutoffs: *P*(*τ*) = *Aτ*^−1−*α*^ exp(−*τ*/*τ_c_*) (*α* = 1.2, on: *τ_c_* = 59 ps, off: *τ_c_* = 1074 ps). (B) Ensemble-averaged PSD of shuffled dichotomous processes (SDP) (black line) on the POPC membrane. Numerical simulation of alternating renewal process; residence times are given by power-law distribution with exponential cutoff, where *α* = 1.2, on: *τ_c_* = 60 ps, off: *τ_c_* = 1000 ps (green line). The solid line is shown for reference.

To confirm a presence of a long-term memory, we calculate the ensemble-averaged PSD for shuffled dichotomous processes, where residence times for “on” and “off” states were shuffled among themselves randomly. Because shuffled dichotomous processes do not have a long-term correlation between residence times, we can clarify the existence of a long-term correlation. The ensemble-averaged PSD of the shuffled dichotomous processes exhibits 1/*f* noise and platuau at low frequencies [Fig. 3B]. However, the power-law exponent of *S*(*f*) ∝ *f*^−*β*^ at high frequencies changes from the original one (1.35) to 0.8. The frequency at which the PSD becomes a plateau is the same order of that of the original dichotomous process in Fig. 2B. This means that the long-term memory in residence times affects the power-law exponents of the original PSD.

Figure 3A shows probability density functions (PDFs) of residence times for “on” and “off” states. Both PDFs follow power-law distributions with exponential cutoffs, *P*(*τ*) = *Aτ*^−1−*α*^ exp(−*τ*/*τ_c_*), where the power-law exponent is *α* = 1.2, and cutoffs for the PDFs of the “on” and “off” states are *τ_c_* = 59 ps and 1074 ps, respectively. The observed exponent, *α* > 1, implies that mean residence time does not diverge and is consistent with the ergodic behavior (no aging). Following our observations, we performed a numerical simulation in which time series of “on” and “off” states were generated with random waiting times drawn from a power-law distribution with an exponential cutoff (*α* = 1.2, on: *τ_c_* = 60 ps, off: *τ_c_* = 1000 ps). The PSD of the numerical simulation is well consistent with that of the shuffled dichotomous process [see Fig. 3B]. In alternating renewal process, the power-law exponent *β* in the PSD is given by the power-law exponent in the residence time distribution, *i.e.*, *β* = 2 − *α* as *α* < 2^15^. The power-law exponent *β* observed here in the PSD is consistent with this relationship.

**Figure 4:**
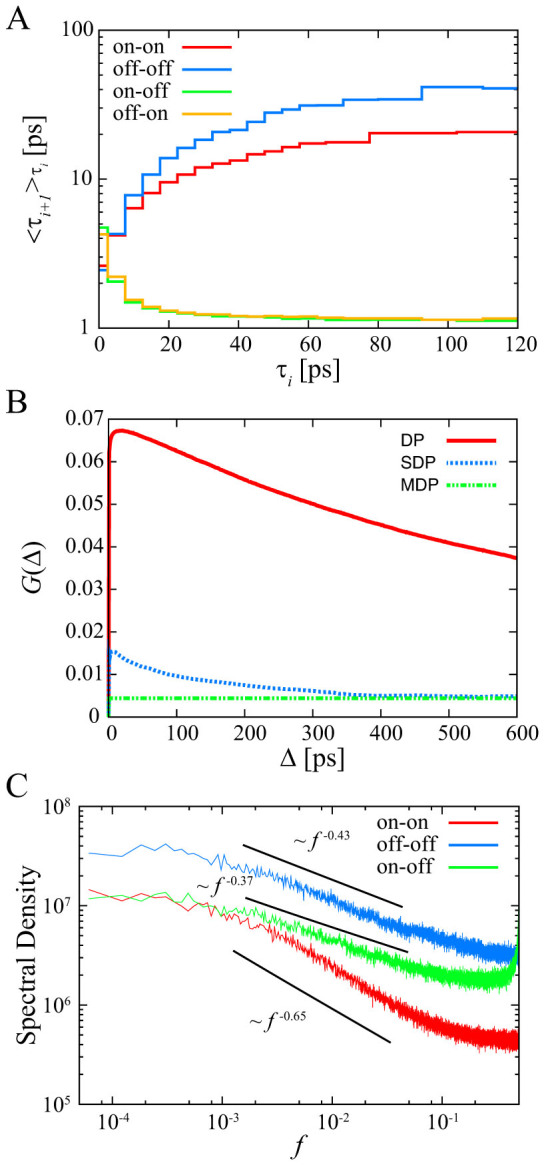
Correlation between residence times on the POPC membrane. (A) Conditional averages of the residence times. Different color lines distinguish the pairs used for the analysis. (B) Degree of non-Markovianity for dichotomous processes (DP), shuffled dichotomous processes (SDP), and Markovian dichotomous processes (MDP). (C) Ensemble-averaged PSD of residence times. The solid lines are shown for reference.

To clarify the correlation between residence times, we consider three types of time series of residence times: 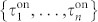, 
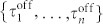
, and 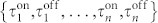. Figure 4A shows the conditional averages of *τ_i_*_+1_, denoted by 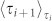, when the previous residence time is in 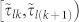 for some 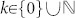, where we set *l* = 10^3^ and 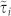 is rearranged in ascending order 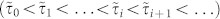
43. There are positive correlations of residence times between the previous “on” state and the current “on” state or the previous “off” state and the current “off” state, and negative correlations of residence times between an “on” state residence time and the next “off” state time or an “off” state residence time and the next “on” state time. This means that each state is stable, *i.e.* the hydration layer is stabilized. Furthermore, we show the degree of non-Markovianity, *G*(Δ, *T*), in Fig. 4B^8^ (the details are shown in supporting information). We used 12800 data at each time step to calculate the transition probabilities, and *G*(Δ, *T*) were calculated for *T* = 600 ps. Maximal values for dichotomous process (DP) and shuffled dichotomous process (SDP) are *G*_DP_(21, 600) = 0.067 and *G*_SDP_(6, 600) = 0.015, respectively. The value for computer-generated Markovian dichotomous processes is *G* = 0.004. The dichotomous process generated by the hydration dynamics shows strong non-Markovianity, while the shuffled dichotomous process also shows non-Markovianity before 400 ps. Moreover, the ensemble-averaged PSDs of the three types of time series of residence times exhibit 1/*f* noise [Fig. 4C]. This result means that the residence times have a long-term correlation. These results imply that the high non-Markovianity of the dichotomous processes comes from not only a power-law residence time distribution but also a long-term memory in residence times. These results suggest that the origin of the 1/*f* noise is a combination of a power-law residence time distribution and a long-term correlation between residence times. These correlations between residence times are also observed in quantum dot blinking experiments^44^,^45^.

## Discussion

Using all-atom molecular dynamics simulations, we have found that fluctuations of number of water molecules on the lipid molecules exhibit 1/*f^β^* noise with *β* > 1 and that the power spectrum does not break ergodicity. Moreover, we have provided an evidence that the 1/*f* noise and ergodic behavior are caused by non-Markov power-law intermittency with exponential cutoff. What is a biological significance of 1/*f* noise in hydration dynamics on lipid membrane surfaces? The roles played by the water molecules near the membrane depend upon their structure and dynamics. There are positive correlations of residence times between the same states, and negative correlations of residence times between the different states. This means that each state is stable, *i.e.* the hydration layer is stabilized. The 1/*f* noise attributed to a correlated renewal process can contribute to the stability of the hydration layer, which is important for membrane stability and physiological processes. Moreover, these results are relevant to a broad range of systems displaying 1/*f* fluctuations.

Because dynamics of lipid molecules and membrane structures affect the hydration dynamics of water molecules, the complexity of lipid membrane surfaces, diffusivity, and fluctuations of lipid height will contribute to the 1/*f* noise. Here we confirmed that temporal fluctuations of the height of lipid molecules also show 1/*f* noise [see Fig. S2]. The transition frequency in the PSD from 1/*f* noise to a plateau is almost the same as that of fluctuation of number of water molecules around the lipid molecule [see Fig. 1B and S2]. It has been shown that lipid bilayers exhibit transient subdiffusion originated from fractional Brownian motion (FBM) (viscoelasticity)^46^,^47^,^48^ and shows dynamic heterogeneity^46^. Moreover, water molecules on the lipid membrane surfaces exhibit subdiffusion, which originates from a combination of long-term correlated noise (FBM) and divergent mean trapping time (continuous-time random walk: CTRW)^36^. A power-law waiting time distribution, arising from random binding of water molecules with the lipid molecules or 1D comb-like structure of lipid membrane surfaces, contributes to CTRW. Viscoelasticity of lipid bilayers contributes to the FBM of the water molecules. Furthermore, the hydrogen-bond exchange dynamics shows long range correlations between multiple water molecules^49^,^50^. These effects will contribute to the origin of the observed 1/*f* noise.

Although we used conventional hydrogen bond distance 0.35 nm to define the surface water molecules, it was shown that there is no preferential mutual orientation between two water molecules if the distance is more than 0.3 nm^49^,^50^. We confirmed 1/*f* noise with different definition of hydrogen bond distance 0.3 nm [see Fig. S3]. The value of the power-law exponents of PSD become *β* = 1.2 at high frequencies. And transition frequency of the PSD shift to lager frequency because the cutoff of the distribution of the residence time in dichotomous process becomes short.

Moreover, surface water structures and diffusivities of lipid molecules are known to be affected by negatively charged lipids and ions^51^,^52^. To clarify the universality of observed 1/*f* noise, we performed additional MD simulations of (i) pure palmitoyl-oleoyl-phosphatidylethanolamine (POPE) membrane [see Fig. S4–S6], (ii) negatively charged membrane of POPC/palmitoyloleoyl phosphatidylserine (POPS) (4:1) lipids with 150 mM NaCl ions [see Fig. S7–S8], and (iii) different force field, thermostat, and barostat [see Fig. S9]. Power-law exponent *β* of PSDs and cutoff time *τ* in the power-law residence time are slightly different. However, there are no significant qualitative differences, that is, the power-law exponent *β* and the origin of the 1/*f* noise [see Fig. S3–S9].

## Methods

### Molecular dynamics simulations

Molecular dynamics (MD) simulations of pure palmitoyl-oleoyl-phosphocholine (POPC) bilayers was performed to clarify the hydration dynamics on the lipid membrane surface. The lipid bilayer system of pure POPC lipids was consisted of 128 lipids (64 for each leaflet) and 7680 TIP3P water molecules. The CHARMM36^53^ force field was used for the lipids. The TIP3P water model modified for the CHARMM force field^54^ was used because the CHARMM36 force field was developed based on it. Although the diffusion constant of the TIP3P water model is higher than the experimental values, it reproduces the first-shell hydration and the energetics of liquid water^55^. The bond lengths involving the hydrogen atoms were constrained to equilibrium lengths using the SHAKE method^56^. The direct sum and Lennard-Jones interactions were smoothly truncated at a cutoff distance of 1.2 nm, using a switching function that becomes effective at 1.0 nm. The particle-mesh Ewald method^57^ was used to calculate electrostatic interactions. Before the MD simulation, energy minimization was performed using a conjugate gradient algorithm to remove any bad contacts from within the initial configuration. The simulation was performed under constant NPT (number of particles, pressure, and temperature) at temperature 310 K and pressure 0.1 MPa. For temperature and pressure control, a Langevin thermostat and piston^58^,^59^ were used with a damping coefficient of 1 ps^−1^ and a collision period of 0.2 ps. The three orthogonal dimensions of the periodic cell were allowed to change independently in the *x*-*y* and *z* dimensions (semi-isotropic pressure coupling). The simulation was performed for 240 ns under 2.0 fs time-step increments; coordinates were recorded every 1.0 ps. The trajectories for the final 131 ns were used for analysis. The MD simulation was performed using NAMD2.9 software^60^. In the main text, we shows the results of the CHARMM force field. The other simulation conditions are shown in supplementary information.

### Degree of non-Markovianity

The degree of non-Markovianity^8^ is given by
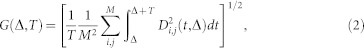
where *M* is the total number of the states (*M* = 2 in our case), *T* is a measurement time, and
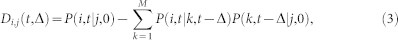
where *P*(*i*, *t*|*j*, *s*) is the transition probability that the current state at the time *t* is in the state number *i* under the earlier state at the time *s* was in the state number *j*. The value of *G* strongly depends on the number of ensemble for calculating the transition probabilities. For calculating the transition probability, 128 dichotomous processes were divided into 100 segments. Thus, we used 12800 data at each time step for calculating the conditional probabilities. The shuffled dichotomous processes were generated by shuffling the residence times for “on” and “off” states among themselves randomly. We generated the Markov dichotomous process where each state is generated independently with equal probability (*p* = 1/2). The *P*(*i*,*t*|*j*, *s*) for the Markov dichotomous process was calculated by using the same number of ensemble 12800. All *G*(Δ, *T*) were calculated for *T* = 600 ps.

## Author Contributions

E.Y. and T.A. performed the calculation and analysis. The research reported here emerged from lively discussions between E.Y., T.A., M.Y. and K.Y. All authors contributed to write the manuscript.

## Supplementary Material

Supplementary Information Supplementary Information
