# Supplementary material for: Origin of 1/f noise in hydration dynamics on lipid membrane surfaces
Source: Sci Rep. 2015 Mar 6;5:8876. doi: 10.1038/srep08876 (PMC4351557; doi:10.1038/srep08876)
Supplement: Supplementary Information [file srep08876-s1.pdf]

Supporting Information for "Origin of $1/f$ noise in hydration dynamics on lipid membrane surfaces"

Eiji Yamamoto,[1] Takuma Akimoto,[1] Masato Yasui,[2] and Kenji Yasuoka[1]

[1] *Department of Mechanical Engineering, Keio University, Yokohama, Japan*

[2] *Department of Pharmacology, School of Medicine, Keio University, Shinjuku-ku, Tokyo, Japan*

Additional molecular dynamics simulations

Molecular dynamics (MD) simulations of pure palmitoyl-oleoyl-phosphatidylethanolamine (POPE) and POPC/palmitoyl-oleoyl phosphatidylserine (POPS) bilayers were performed to clarify the universality of the hydration dynamics on the lipid membrane surfaces. The CHARMM36 [1] force field was used for the lipids. The lipid bilayer systems of pure POPE lipids consisted of 128 lipids (64 for each leaflet) and 7,680 TIP3P water molecules. The POPC/POPS (4:1) lipid system of 100 POPC lipids and 28 POPS lipids was solvated with 7552 TIP3P water molecules, and NaCl ions at 150 mM concentration were added to neutralize the system. The simulation conditions were the same as that in the main text.

To confirm the universality of results, we changed water models, force fields, thermostat, and barostat. We performed a 1.14 μs MD simulation of the membrane system which has 128 POPC molecules and 7823 SPC water molecules [2] using GROMACS-4.5.5 software [3]. The GROMOS96 53a6 force field [4], which is a united-atom model, was used for the POPC lipid. The pressure of 1 bar and a temperature of 310 K were controlled using the Parrinello-Rahman barostat [5] and velocity rescaling method [6] with a coupling time of 1 ps and 0.1 ps, respectively. The lengths of bonds involving the hydrogen atoms were constrained to equilibrium lengths using the LINCS method [7]. The time step was set at 2 fs. The particle-mesh Ewald method was used for long-range electrostatic interactions. A cut-off distance of 1 nm was used for the van der Waals interactions.

---

[1] Klauda, J. B. *et al.* Update of the charmm all-atom additive force field for lipids: validation on six lipid types. *J. Phys. Chem. B* **114**, 7830–7843 (2010).

[2] Berendsen, H. J. C., Postma, J. P. M., Van Gunsteren, W. F. & Hermans, J. Interaction models for water in relation to protein hydration. In *Intermolecular forces*, 331–342 (Springer, 1981).

[3] Hess, B., Kutzner, C., Van Der Spoel, D. & Lindahl, E. Gromacs 4: Algorithms for highly efficient, load-balanced, and scalable molecular simulation. *J. Chem. Theory Comput.* **4**, 435–447 (2008).

[4] Oostenbrink, C., Villa, A., Mark, A. E. & Van Gunsteren, W. F. A biomolecular force field based on the free enthalpy of hydration and solvation: The gromos force-field parameter sets 53a5 and 53a6. *J. Comput. Chem.* **25**, 1656–1676 (2004).

[5] Parrinello, M. & Rahman, A. Polymorphic transitions in single crystals: A new molecular dynamics method. *J. Appl. Phys.* **52**, 7182–7190 (1981).

[6] Bussi, G., Donadio, D. & Parrinello, M. Canonical sampling through velocity rescaling. *J. Chem. Phys.* **126**, 014101 (2007).

[7] Hess, B., Bekker, H., Berendsen, H. J. C. & Fraaije, J. G. E. M. Lincs: a linear constraint solver for molecular simulations. *J. Comput. Chem.* **18**, 1463–1472 (1997).

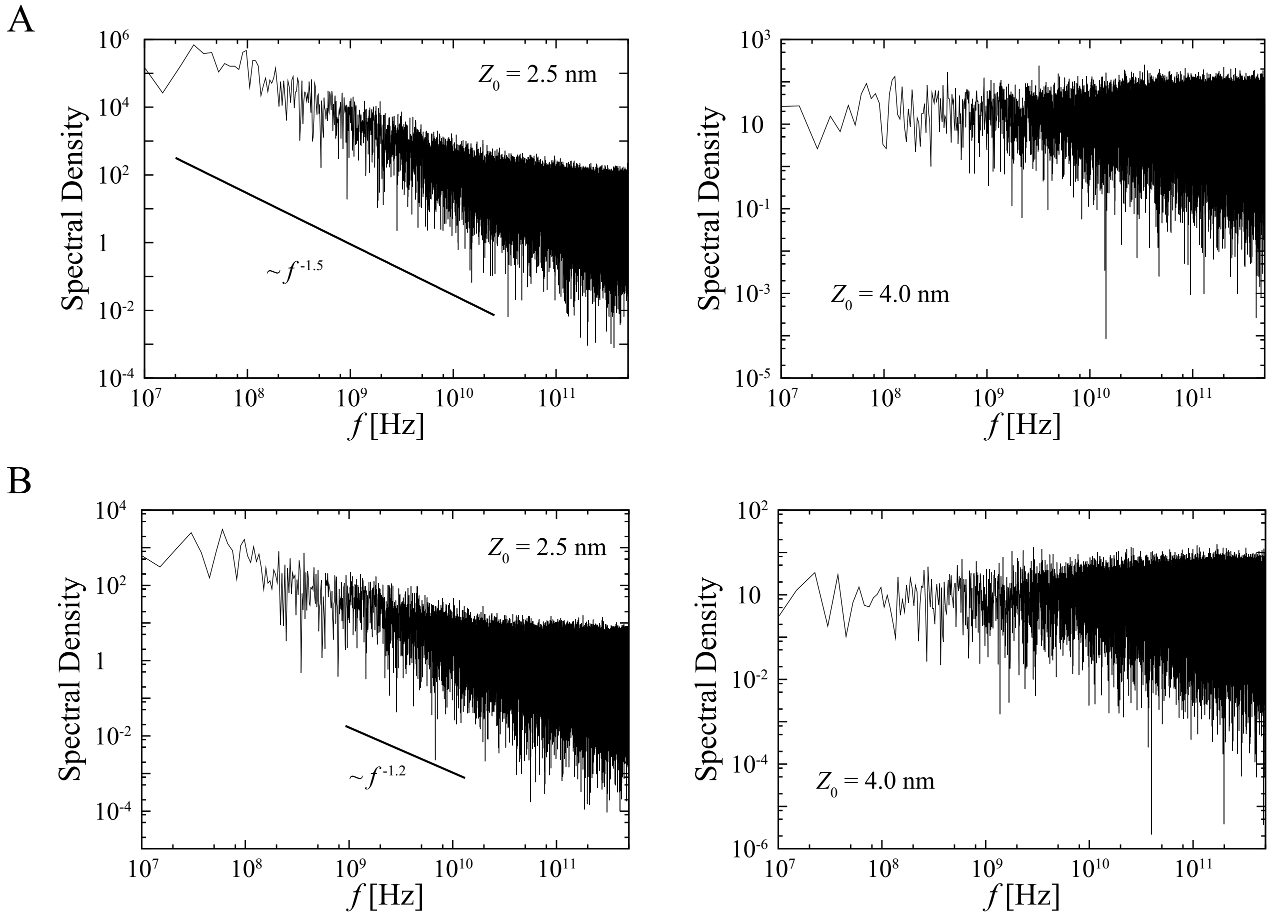

Fig. S 1: Ensemble-averaged PSD of number of water molecules in regions near the POPC membrane surface. (A) Box (size is $l_x \times l_y \times l_z = 3 \times 3 \times 0.7$ nm^3) and (B) sphere (radius is $r = 0.35$ nm^3). The centers of the box and sphere are located at a perpendicular diastase Z_0 from the center of mass of the membrane. $Z_0 = 2.5$ nm is around the surface of the membrane. The fluctuations at $Z_0 = 4.0$ nm are white Gaussian noise.

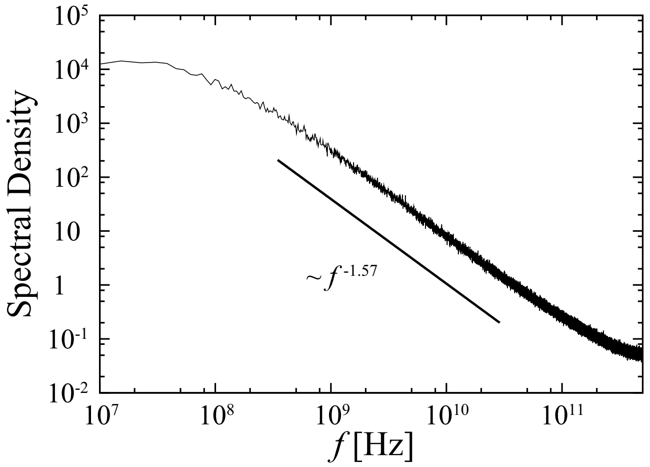

Fig. S 2: Ensemble-averaged PSD of temporal fluctuations of perpendicular position of lipid molecules in the POPC membrane. The position of a lipid molecule is defined by the perpendicular distance between center of mass of the membrane and a phosphorus atom in the phosphate group of the lipid molecule. We use 128 time series (128 lipid molecules) to obtain the ensemble-averaged PSD. The PSD exhibits $1/f$ noise.

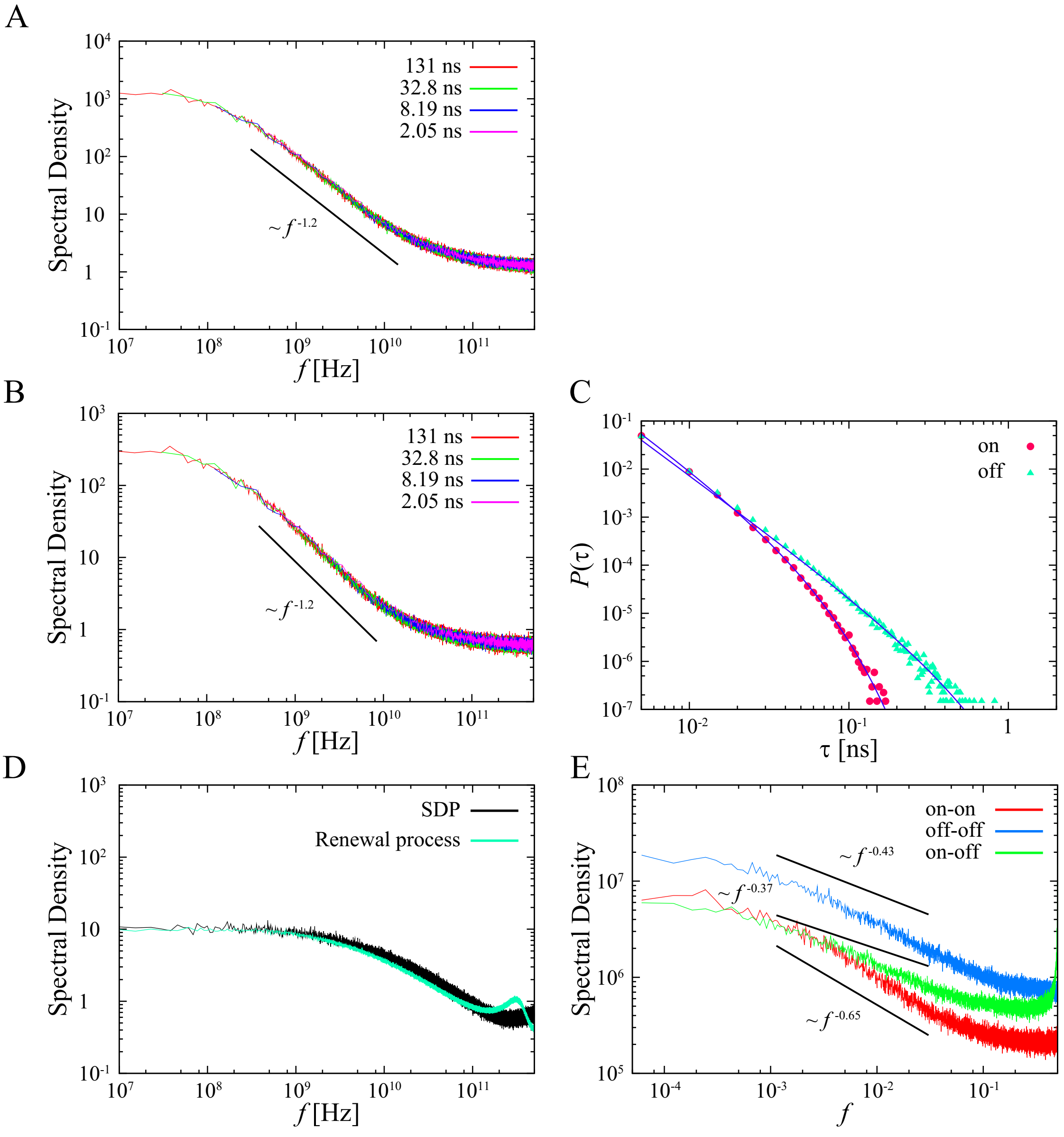

Fig. S 3: $1/f$ noise in the hydration dynamics on the POPC membrane with using different hydrogen bond distance 0.3 nm. (A) Ensemble-averaged PSD of number of water molecules on POPC lipid head group. We use 128 time series to obtain the ensemble-averaged PSD. The different colored lines represent different measurement times. The solid line is shown as a reference. (B) Ensemble-averaged PSD of the time series of the two states. The dichotomous process was generated in the same way as the POPC membrane in the main text. (C) PDFs of residence times of "on" and "off" states on POPC. Solid lines are fitted curves for power-law distributions with exponential cutoffs: $P(\tau) = A\tau^{-1-\alpha}\exp(-\tau/\tau_c)$ ($\alpha = 1.4$, on: $\tau_c = 35$ ps, off: $\alpha = 1.45$, $\tau_c = 350$ ps). (D) Ensemble-averaged PSD of shuffled dichotomous processes (SDP) (black line). Numerical simulation of alternating renewal process; residence times are given by power-law distribution with exponential cutoff, where on: $\alpha = 1.4$, $\tau_c = 35$ ps, off: $\alpha = 1.45$, $\tau_c = 350$ ps (green line). (E) Ensemble-averaged PSD of residence times of two states. There are no significant qualitative differences in observation of $\beta \geq 1$ and the origin of the $1/f$ noise compared to the definition of the hydrogen bond distance 0.35 nm.

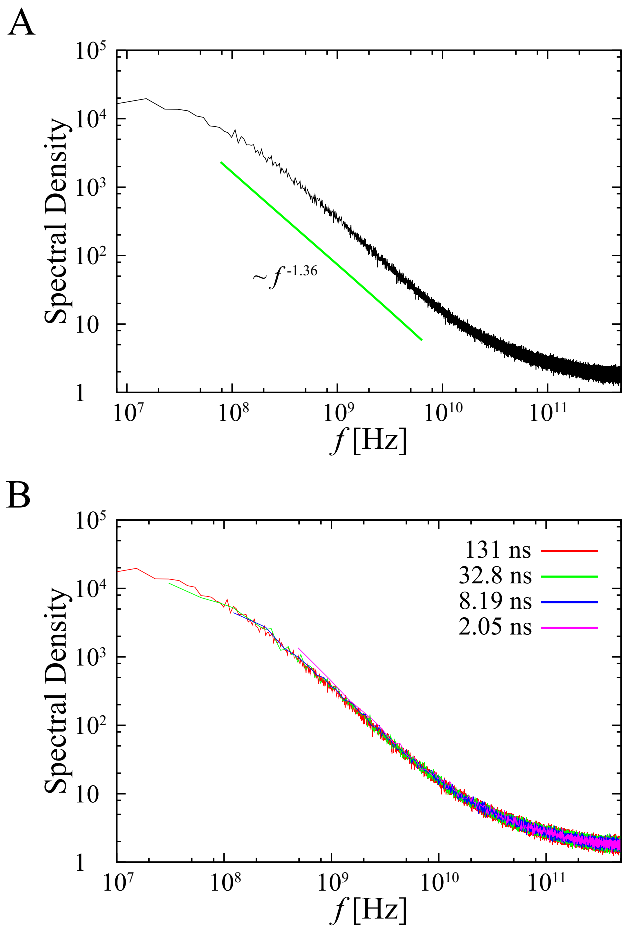

Fig. S 4: $1/f$ noise in the hydration dynamics on the POPE membrane. (A) Ensemble-averaged PSD of number of water molecules on a lipid head group. We use 128 time series to obtain the ensemble-averaged PSD. The solid lines represent power-law behavior for reference. Total measurement time was 131 ns. (B) Ensemble-averaged PSD for four different measurement times: 2.05, 8.19, 32.8, and 131 ns. There is no aging.

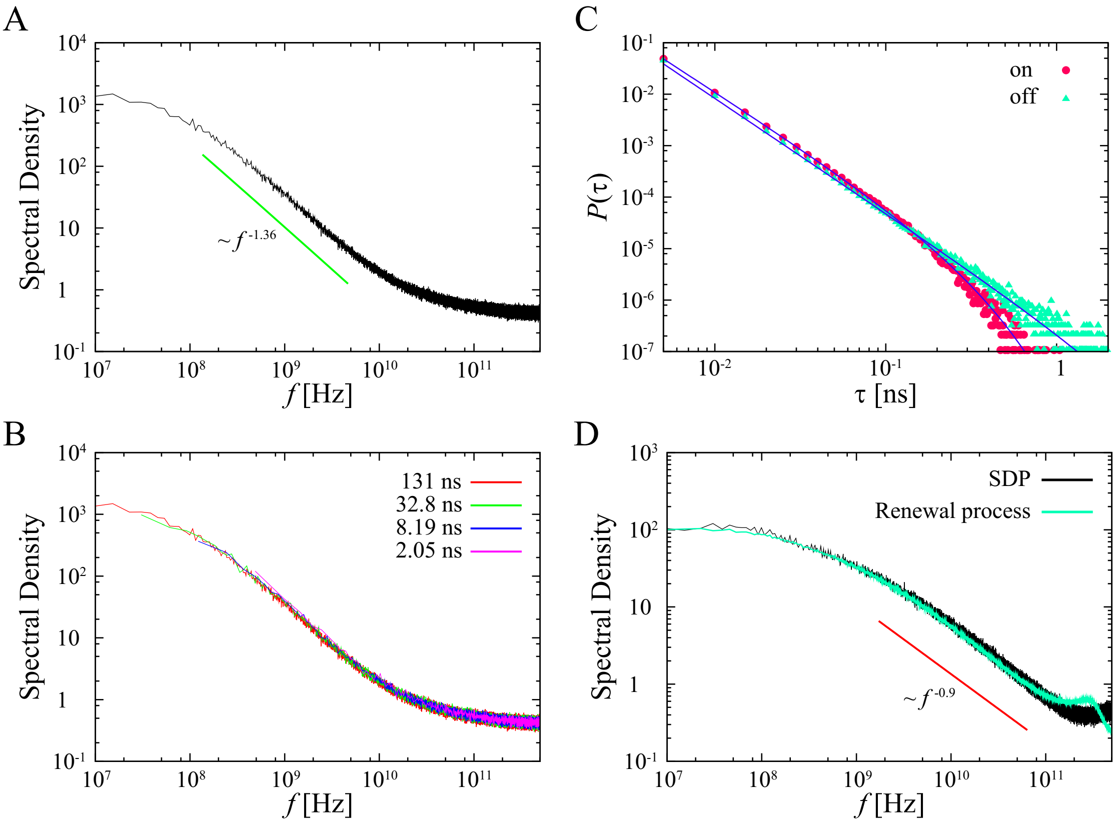

Fig. S 5: $1/f$ noise in the dichotomous process on the POPE membrane. The dichotomous process was generated in the same way as the POPC membrane in the main text. (A) Ensemble-averaged PSD of the time series of the two states. We use 128 time series to obtain the ensemble-averaged PSD. The solid lines are shown as reference for higher and lower frequencies. (B) Ensemble-averaged PSD of the time series of the two states for four different measurement times: 2.05, 8.19, 32.8, and 131 ns. There is no aging. (C) PDFs of residence times of "on" and "off" states. Solid lines are fitted curves for power-law distributions with exponential cutoffs: $P(\tau) = A\tau^{-1-\alpha}\exp(-\tau/\tau_c)$ (on: $\alpha = 1.1$, $\tau_c = 240$ ps, off: $\alpha = 1.2$, $\tau_c = 3000$ ps). (D) Ensemble-averaged PSD of shuffled dichotomous processes (SDP) (black line). Numerical simulation of alternating renewal process; residence times are given by power-law distribution with exponential cutoff, where on: $\alpha = 1.1$, $\tau_c = 240$ ps, off: $\alpha = 1.2$, $\tau_c = 3000$ ps (green line). The solid line is shown for reference.

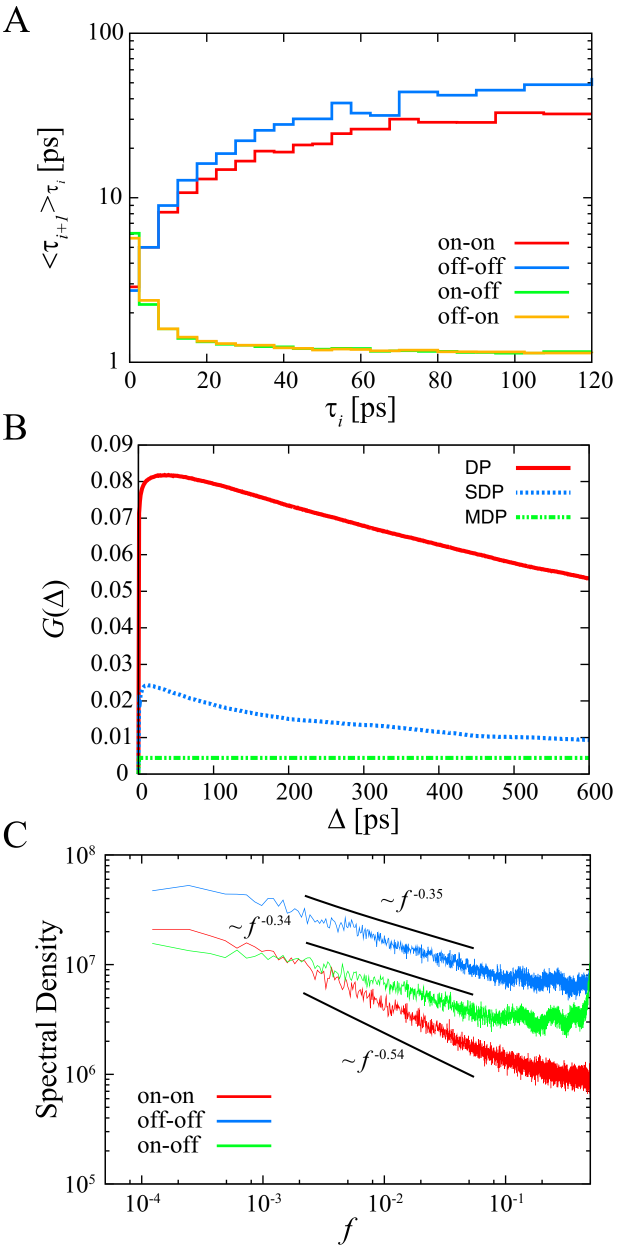

Fig. S 6: Correlation of residence times on the POPE membrane. (A) Conditional averages of the residence times. Different color lines distinguish the pairs used for the analysis. (B) Degree of non-Markovianity for dichotomous processes (DP), shuffled dichotomous processes (SDP), and Markovian dichotomous processes (MDP). (C) Ensemble-averaged PSD of residence times. The solid lines are shown for reference.

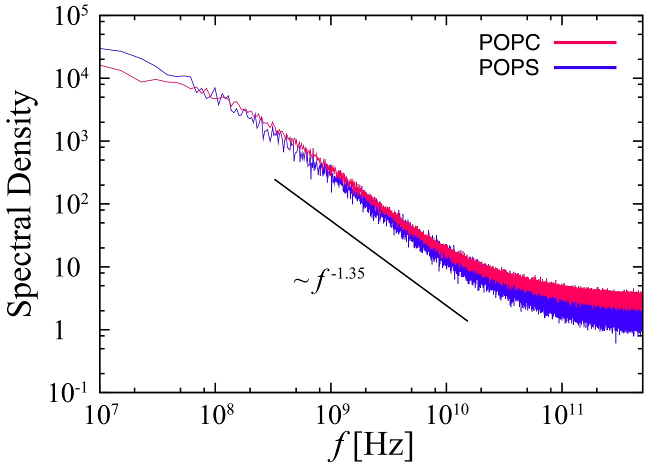

Fig. S 7: $1/f$ noise in the hydration dynamics on the POPC/POPS membrane. Ensemble-averaged PSD of number of water molecules on a lipid head groups. We use 100 and 28 time series to obtain the ensemble-averaged PSDs on POPC (red) and POPS (blue) lipids, respectively. The solid lines represent power-law behavior for reference. Total measurement time was 131 ns. We confirmed that there is no aging.

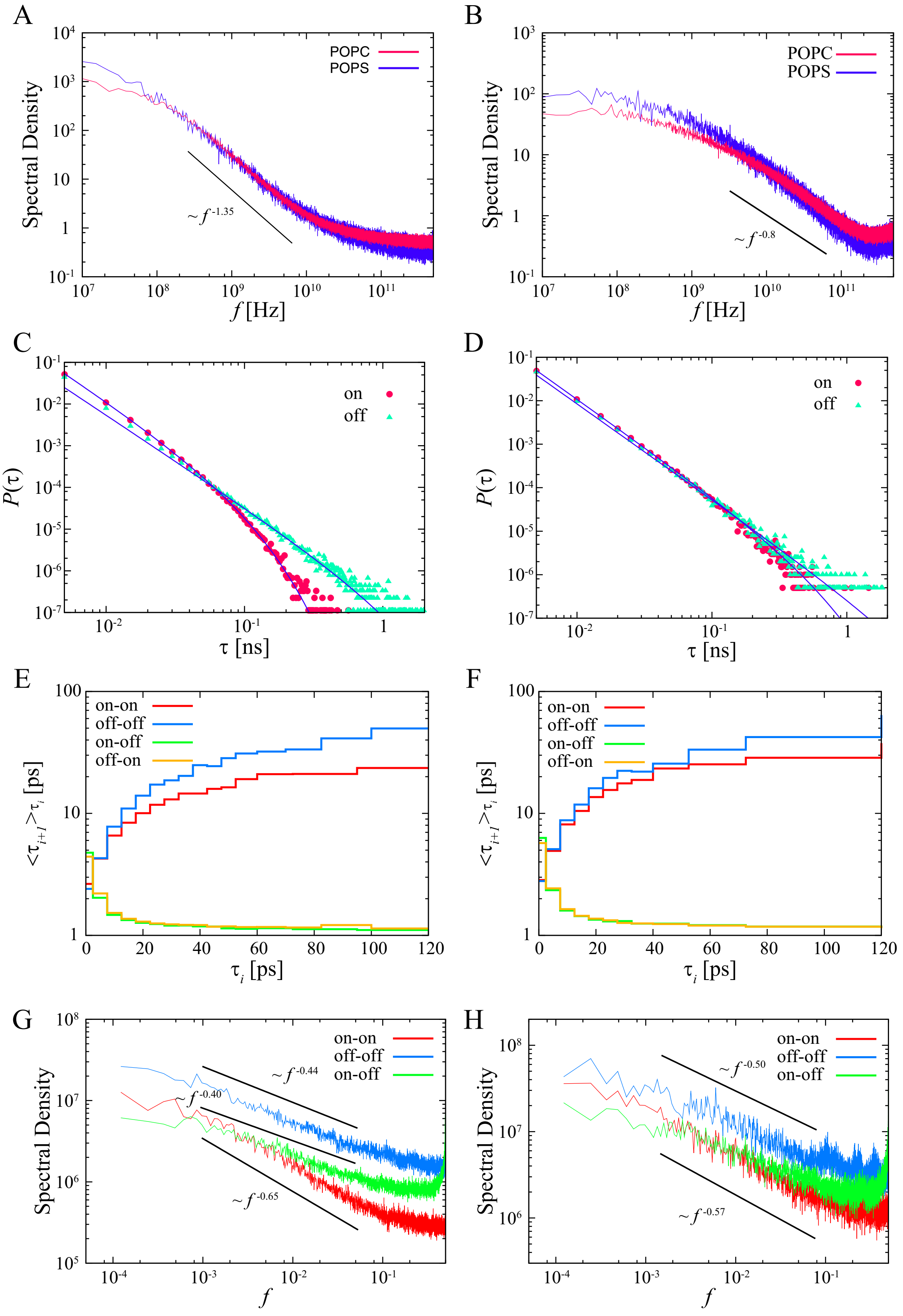

Fig. S 8: $1/f$ noise in the dichotomous process on the POPC/POPS membrane. The dichotomous process was generated in the same way as the POPC membrane in the main text. (A) Ensemble-averaged PSD of dichotomous processes . We use 100 and 28 time series to obtain the ensemble-averaged PSD on POPC and POPS lipids, respectively. The solid lines are shown as reference for higher and lower frequencies. We confirmed that there is no aging (results are not shown). (B) Ensemble-averaged PSD of shuffled dichotomous processes. (C) PDFs of residence times of "on" and "off" states on POPC. Solid lines are fitted curves for power-law distributions with exponential cutoffs: $P(\tau) = A\tau^{-1-\alpha}\exp(-\tau/\tau_c)$ ($\alpha = 1.2$, on: $\tau_c = 68$ ps, off: $\tau_c = 1000$ ps). (D) PDFs of residence times of "on" and "off" states on POPS. Solid lines are fitted curves for power-law distributions with exponential cutoffs: $P(\tau) = A\tau^{-1-\alpha}\exp(-\tau/\tau_c)$ ($\alpha = 1.2$, on: $\tau_c = 513$ ps, off: $\tau_c = 3420$ ps). (E) Conditional averages of the residence times on POPC and (F) POPS lipids. (G) Ensemble-averaged PSD of residence times on POPC and (H) POPS lipids. The solid lines are shown for reference.

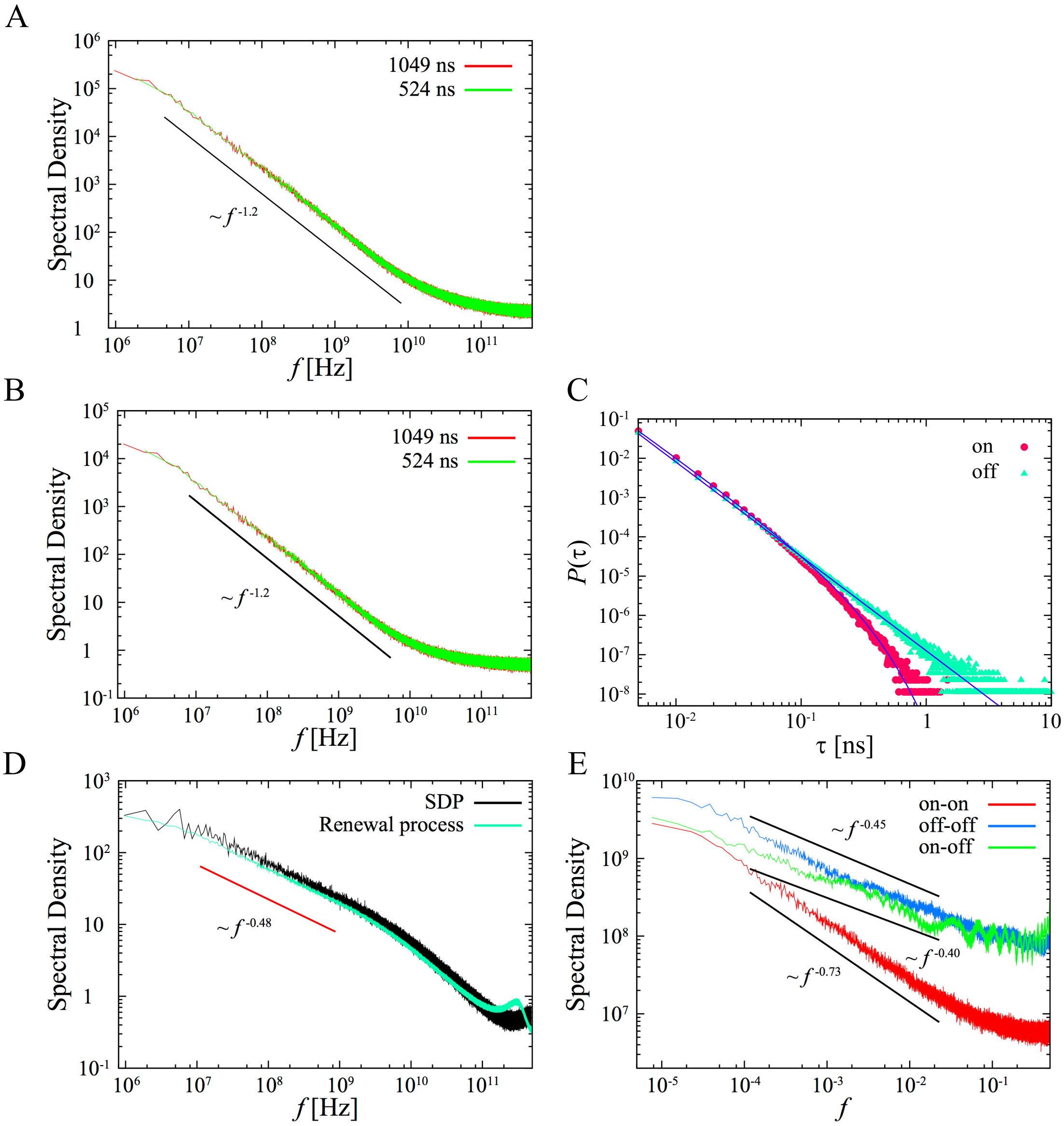

Fig. S 9: $1/f$ noise in the hydration dynamics on the POPC membrane with using GROMOS force filed, SPC water model, Parrinello-Rahman barostat and velocity rescaling method for temperature controlling. (A) Ensemble-averaged PSD of number of water molecules on POPC lipid head group. We use 128 time series to obtain the ensemble-averaged PSD. The different colored lines represent different measurement times. The solid line is shown as a reference. (B) Ensemble-averaged PSD of the time series of the two states. The dichotomous process was generated in the same way as the POPC membrane in the main text. (C) PDFs of residence times of "on" and "off" states on POPC. Solid lines are fitted curves for power-law distributions with exponential cutoffs: $P(\tau) = A\tau^{-1-\alpha}\exp(-\tau/\tau_c)$ ($\alpha = 1.3$, on: $\tau_c = 196$ ps, off: $\alpha = 1.4$, $\tau_c = 100000$ ps). (D) Ensemble-averaged PSD of shuffled dichotomous processes (SDP) (black line). Numerical simulation of alternating renewal process; residence times are given by power-law distribution with exponential cutoff, where on: $\alpha = 1.3$, $\tau_c = 200$ ps, off: $\alpha = 1.4$, $\tau_c = 100000$ ps (green line). (E) Ensemble-averaged PSD of residence times of two states.
